# Measurement of Fall Prevention Awareness and Behaviours among Older Adults at Home*

**DOI:** 10.1017/S0714980817000332

**Published:** 2017-12

**Authors:** Katherine Russell, Darcie Taing, Jacqueline Roy

**Affiliations:** 1 Ottawa Public Health

**Keywords:** aging, community dwelling, fall prevention, awareness, public health, vieillissement, vivant dans la communauté, prévention des chutes, sensibilisation, santé publique

## Abstract

This study surveyed awareness of, and adherence to, six national fall prevention recommendations among community-dwelling older adults (*n* = 1050) in Ottawa. Although 76 per cent of respondents agreed falling is a concern and preventable, fewer perceived susceptibility to falling (63%). Respondents had high awareness that home modifications and physical activity can prevent falls. Reported modifications included grab bars (50%), night lights (44%), and raised toilet seats (19%). Half met aerobic activity recommendations; 38 per cent met strength recommendations. Respondents had lower awareness that an annual medication review, annual eye and physical examination, and daily vitamin D supplementation could reduce fall risk. However, reported annual medication review (79%) and eye examination (75%) was high. Nearly half met recommendations for vitamin D intake. These findings suggest a gap in knowledge of awareness and adherence to national recommendations, highlighting the ones that may require attention from those who work to prevent falls.

## RÉSUMÉ

L’étude visait à évaluer chez les résidents aînés d’Ottawa (n = 1 050) la connaissance et le respect de six recommandations nationales sur la prévention des chutes. D’abord, 76 % des répondants étaient d’avis que les chutes sont une préoccupation réelle et qu’il est possible de les éviter, mais ils étaient moins nombreux (63 %) à penser qu’ils étaient à risque de faire une chute. Ensuite, les répondants étaient très conscients que l’activité physique et l’adaptation du domicile permettent de prévenir les chutes. Parmi les adaptations mentionnées figuraient les barres d’appui (50 %), l’éclairage de nuit (44 %) et les sièges de toilette surélevés (19 %). En ce qui concerne l’activité physique, la moitié des répondants suivaient les recommandations visant à améliorer leur capacité cardiovasculaire, et 38 %, celles visant à améliorer leur force. Enfin, les répondants étaient moins sensibilisés à l’importance de la vérification annuelle des médicaments, de l’examen médical et de l’examen de la vue annuels et de la prise quotidienne de suppléments de vitamine D pour la réduction des risques de chute. Cependant, ils étaient nombreux à indiquer qu’ils faisaient vérifier leurs médicaments (79 %) et passaient un examen de la vue (75 %) tous les ans. De plus, près de la moitié respectaient les recommandations sur la prise de suppléments de vitamine D. Ces résultats font ressortir un certain écart entre la connaissance et le respect des recommandations nationales, mettant en évidence celles qui mériteraient l’attention des professionnels œuvrant dans la prévention des chutes.

Although largely preventable, falls represent a tremendous health and economic burden across Canada, particularly among older adults. Falls among older adults, aged 65 years and older, accounted for $3.4 billion (direct and indirect costs) nationally in 2010, accounting for 39 per cent of the total cost of falls in Canada (Parachute, 2015).

In Ottawa, falls are the leading cause of injury-related emergency room visits, hospitalization, and death among older adults. Every year, approximately one fifth of older Ottawa adults who live in private homes fall (Ottawa Public Health [OPH], 2015f), and those falls contribute to more than 8,200 emergency room visits (OPH, 2015c), 2,100 hospitalizations (OPH, 2015a), and approximately 90 deaths (OPH, 2015b). This will continue to be a public health concern as Ottawa’s population aged 65 years and older is the fastest growing age group and is predicted to grow from 15 per cent (140,136) in 2015 to 21 per cent (247,973) in 2030 (OPH, 2015d). Of particular concern are adults aged 85 years and older, a group that has significantly higher rates of falls and the highest cost per capita for falls (OPH, 2015a, 2015c; Parachute, 2015).

Public health contributes to fall prevention using a multifaceted population health promotion approach. This type of approach focuses on improving the health status of an entire population, including groups within it (Hamilton & Bhatti, 1996). This means that public health invests in the surveillance and reporting of the burden of falls, the uptake of fall prevention strategies, health education and public awareness campaigns on fall risk and prevention, and partnerships with different sectors who play a role in the prevention or treatment of falls or those who work with populations most at-risk of falling. The Public Health Agency of Canada (2014) and others (Scott, Dukeshire, & Gallagher, 2001; Scott, 2012) have provided several recommendations for fall prevention in community settings. Those that fit within public health’s role include promoting (1) a review of medications annually with a physician or pharmacist; (2) an annual medical examination; (3) an annual vision examination; (4) the accumulation of at least 150 minutes of moderate- to vigorous-intensity aerobic physical activity per week including strength and balance activities at least two days per week according to Canada’s Physical Activity Guidelines for Older Adults (CPAG-OA) (Canadian Society for Exercise Physiology, 2015); (4) proper nutrition including adequate vitamin D and calcium intake according to Canada’s Food Guide for older adults (Health Canada, 2012); and (5) the identification and removal of home hazards and the installation and use of home safety devices.

## Gaps in Local Fall Prevention Behaviour Data

There is a gap in local data on the six fall prevention recommendations for Ottawa’s older adult community-dwelling population. Although existing Canadian data sources have measured aspects of fall prevention behaviours, they often assess the general population and are not specific to older adults. They also do not measure the six recommendations concurrently.

Statistics Canada’s (2008) Canadian Community Health Survey on Healthy Aging (CCHS-HA) collected information on Canadian adults aged 45 and older living in private residences about the factors that contribute to healthy aging; however, those data are not reportable at the public health unit level. Statistics Canada’s (2015) Canadian Community Health Survey (CCHS) collects information for the Canadian population aged 12 and older with estimates that are reportable for public health units in Ontario. Although the CCHS includes measures that describe the six behaviours, as a general population survey it would take several years of data collection to ensure sufficient sample size collection to report on these indicators for older adults at the local level, particularly for specific age groups within the older adult population. This is also true for the Rapid Risk Factor Surveillance System (RRFSS), which is an ongoing telephone health survey of adults aged 18 years and older conducted by a number of health units in Ontario that includes questions on fall prevention–related behaviours (Rapid Risk Factor Surveillance System, n.d.).

To address this gap at the local level and inform future programming, OPH designed a survey to measure awareness and uptake of the six fall prevention recommendations among community-dwelling older adults. This article outlines the design of the survey tool and presents key findings of the Ottawa Public Health Older Adults Fall Prevention Survey.

## Methods

### Questionnaire Development

The objective of the survey was to measure awareness and uptake of the six fall prevention recommendations. Several questions were replicated from established surveys, including the CCHS-HA (Statistics Canada, 2008) annual medical examination, the review of medications and vision testing questions, and the RRFSS (n.d.) fall prevention home hazards questions. To measure moderate- to vigorous-intensity physical activity and strength and balance activities, we considered existing physical activity questionnaires (DiPietro, Caspersen, Ostfeld, & Nadel, 1993a; DiPietro, Caspersen, Ostfeld, & Nadel, 1993b; The IPAQ group, n.d.; Mayer, Steinman, Williams, Topolski, & LoGerfo, 2008; Statistics Canada, 2011; Washburn, Smith, Jette, & Janney, 1993). The Community Healthy Activities Model Program for Seniors (CHAMPS) Physical Activity Questionnaire for Older Adults (University of California, San Francisco Institute for Health & Aging, 2008) was selected because we could use it to measure the frequency and duration of specific activities, including those related to strength, balance, and flexibility. The questions were activity-specific, which we could score with a metabolic equivalent (MET) value to specifically measure moderate- to vigorous- activity. The questionnaire was also relatively simple to administer, and the tool has been recommended for use in self-report physical activity measurement among older adults (Falck, McDonald, Beets, Brazendale, & Liu-Ambrose, 2016; Stewart et al., 2001). We modified the CHAMPS questions for telephone use and for seasonal activities appropriate to older adults living in Ottawa. To assess the frequency of vitamin D and calcium supplementation and calcium-rich food intake, we modified questions from the CCHS-HA to measure the frequency of vitamin D and calcium vitamin and supplement intake over the past 30 days. Five new questions were developed to measure intake of common calcium-rich foods (milk, hard cheese, yogurt, fortified orange juice, and canned fish with bones). We also developed questions to measure awareness of each of the six prevention recommendations. See Table 1 for a list of the prevention behaviours, indicators, and existing questionnaires used to develop the questionnaire.

**Table 1:** Survey objectives, measures, indicators, and question source

Objective: To measure awareness and uptake of falls-prevention behaviours among older Ottawa adults		
Risk Perception and Behaviour	Indicator(s)	Questionnaire Source
1. Perception of the susceptibility and preventability of falls	• The % of older adults who agree that falling is a concern for older adults. • The % of older adults who agree that falls in older adults can be prevented. • The % of older adults who say that taking 4 or more medications daily can increase the risk for falling. • The % of older adults who say that reviewing medications annually can decrease the risk of falling. • The % of older adults who say that having an annual eye examination can reduce the risk of falling. • The % of older adults who say that installing and using home safety devices can reduce the risk of falling. • The % of older adults who say that being active can reduce the risk of falling. • The % of older adults who say that regularly participating in strength or resistance exercise can reduce the risk of falling. • The % of older adults who say that taking a vitamin D supplement daily can reduce the risk of falling.	New questions using 5-point Likert item response for falls risk perception. A 4-point Likert item response was used for knowledge of preventability and modifiable behaviours.
2. Review medications annually with physician or pharmacist	• The % of older adults who are taking 4 or more medications on the same day. • The % of older adults who have an annual review of their medications by a health care professional. • The % of older adults who are taking 4 or more medications on the same day and have had an annual review of their medications by a health care professional.	RRFSS – Falls Prevention – Medication Use.
3. Have an annual medical examination	• The % of older adults who report having an annual medical examination (excluding check-ups during visits for specific health problems).	RRFSS – Access to Clinical Services.
4. Have an annual vision examination	• The % of older adults who report having an annual vision examination.	CCHS – Eye examinations.
5. Accumulate at least 150 minutes of moderate- to vigorous-intensity aerobic physical activity per week including strength and balance activities at least 2 days per week	• The % of older adults accumulating at least 150 minutes of moderate- to vigorous-intensity physical activity per week. • The % of older adults participating in strength and balance activities at least twice per week.	CHAMPS
6. Eat well and include adequate vitamin D and calcium intake	• The % of older adults who take a vitamin D supplement daily. • The % of older adults who take a vitamin D supplement daily and eat at least 3 servings of calcium-rich foods daily or eat at least 2 servings of calcium-rich foods and a vitamin D supplement daily.	CCHS-HA – Dietary supplement use – vitamins and minerals. New questions (frequency of consumption during a typical week).
7. Identify and remove home hazards and install home safety devices	• The % of older adults who have home hazards that can increase the risk of falling. • The % of older adults who use home modifications of hazards to reduce their risk of falling.	RRFSS – Restriction of activities module. RRFSS – Home modifications.

CHAMPS = Community Healthy Activities Model Program for Seniors

CCHS = Canadian Community Health Survey

CCHS-HA = Canadian Community Health Survey on Healthy Aging

RRFSS = Rapid Risk Factor Surveillance System

To minimize bias in the questionnaire design, we pilot-tested the questionnaire on a small group (*n* = 6) of community-dwelling older adult males and females to review the survey length and the clarity of the questions, as well as to identify questions that might be sensitive to answer. Results of the pilot suggested that respondents had a clear understanding of the survey objectives; the length of the survey was appropriate (approximately 20 minutes); answer choices were clear; respondents felt comfortable answering all questions except income; and no items produced irritation, embarrassment, or confusion. We made slight adjustments to more clearly differentiate some of the physical activities such as moderate to heavy house and yard work.

We used the *A pRoject Ethics Community Consensus Initiative* tool (Alberta Innovates Health Solutions, 2015) to determine the risk and appropriate ethics review. The results indicated that the project involved minimal risk to the population and reinforced that the purpose of the project was for quality improvement of our fall prevention program. We followed a verbal consent script and maintained respondent anonymity and confidentiality by collecting and reporting non-identifying aggregate measures.

### Study Design

A stratified random sample of adults aged 65 years and older living in Ottawa was selected by randomly dialing telephone numbers of Ottawa residents and asking if anyone aged 65 years or older and speaking English or French lived there. To allow for analysis by age, three age group samples were collected: ages 65 to 74, 75 to 84, and 85 years and older. The survey was conducted via computer-assisted telephone interview by Nanos Research on behalf of OPH in December 2012. Older adults were excluded if they did not have a landline telephone number, if they did not speak English or French, or if they could not complete or understand the telephone based questionnaire.

### Analysis

To account for the age-stratified design, we generated sampling weights and applied them using 2011 Census population data for Ottawa, representing 116,593 older adults. We followed this up by univariate analysis of the data by gender, age, mother tongue language, immigration, income, and education and calculated coefficients of variation (CV). Estimates were considered reliable for use if the CV was less than 16.6 per cent; estimates where the CV was between 16.6 per cent and 33.3 per cent were interpreted with caution due to the high sampling variability; and estimates with CVs greater than 33.3 per cent were deemed unreliable. All statistical analyses were conducted using Stata SE V.13 using Pearson’s chi-squared tests with α = 0.05 to assess for statistical significance – these *p* values are presented in Tables 3, 4, and 5. We made multiple comparisons between pairs if the overall chi-squared test indicated significance and adjusted them with a Bonferroni correction.

## Results

### Survey Completion

**Figure 1:**
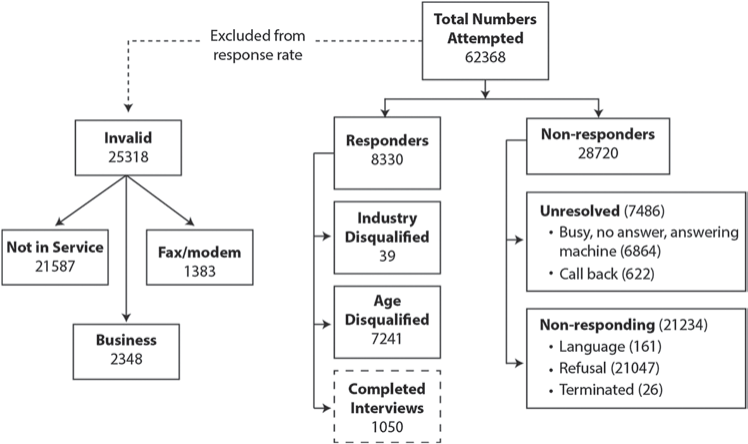
Flowchart of random telephone sampling responses to the survey

As Figure 1 shows, a total of 62,368 telephone numbers were called resulting in 8,330 responders and 28,720 non-responders. Of the responders, we disqualified 7,241 because of their age. A total of 1,050 interviews were completed: 400 for ages 65 to 74, 400 for ages 75 to 84, and 250 for ages 85 years and older. We calculated a response rate of 23 per cent using the empirical method approved by the Marketing Research and Intelligence Association (Marketing Research and Intelligence Association, n.d.), equal to the number of responders divided by the total number of people called.

### Sample Characteristics (unweighted)

The majority of the respondents identified as female (64%). English was the most common mother tongue language (76%) and Canada was the most common country of birth (76%). Household income was evenly distributed across income categories, although 32 per cent of respondents refused to state their income. Fifty five percent of respondents reported that they lived with someone else (Table 2).

**Table 2:** Demographic characteristics of the respondents

Characteristic	No.	%
Gender		
Male	377	35.9
Female	673	64.1
Age (years)		
65 to 74	400	38.1
75 to 84	400	38.1
85 and older	250	23.8
Mother tongue language		
English	800	76.3
French	122	11.6
Other	127	12.1
Income		
Less than $40,000	188	17.9
$40,000 to $69,999	216	20.6
$70,000 or more	224	21.3
Don’t know	86	8.2
Refused to answer	336	32.0
Immigration		
Born in Canada	797	75.9
Immigrant	253	24.1
Length of time since immigration		
≤5 years	1	0.4
6 to 10 years	3	1.2
>10 years	249	98.4
Live with anyone		
Yes	579	55.7
No	461	44.3

### Perceived Susceptibility to Falling

**Figure 2:**
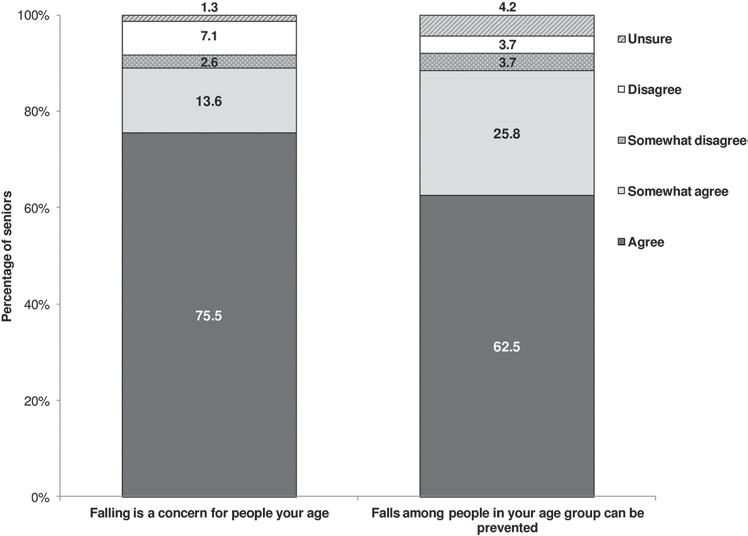
Perceived susceptibility and preventability of falls

When asked if they thought falling was a concern for people their age, 76 per cent agreed and 14 per cent somewhat agreed. When asked if they thought that falls among people in their age group could be prevented, 63 per cent agreed and 26 per cent somewhat agreed (Figure 2). Females, adults aged 85 years and older, and lower income respondents were more likely to agree that falling was a concern for people their age. Respondents with a mother tongue language other than English or French were more likely to agree that falls among their age group could be prevented (Table 3).

**Table 3:** Perceived susceptibility of falling and awareness of falls-prevention behaviours by socio-demographic characteristics (%)

Characteristic	Falling is a Concern for People Your Age (Agree)	Falls among People in Your Age Group Can Be Prevented (Agreed)	Taking ≥4 Medications Daily Increases Risk of Falling	Annual Review of Medication with Health Professional Reduces Risk of Falling	Annual Eye Examination Reduces Risk of Falling	Being Active for at least Two and a Half Hours a Week Reduces Risk of Falling	Regularly Participating in Strength or Resistance Exercises Reduces Risk of Falling	Daily Vitamin D Supplementation Reduces Risk of Falling	Installing and Using Home Safety Devices Reduces Risk of Falling
Ottawa	75.5	62.5	44.0	56.4	77.0	86.4	76.8	53.1	90.0
Gender									
Male	67.7	63.0	42.5	52.8	72.9	86.7	75.3	43.6	89.3
Female	81.5	62.2	45.1	59.1	80.1	86.1	78.0	60.4	90.5
*p* value	0.00	0.55	0.73	0.28	0.00	0.51	0.20	0.00	0.85
Age (years)									
65 to 74	67.4	63.9	52.1	61.9	82.8	90.8	83.0	52.9	92.1
75 to 84	83.9	59.2	38.7	53.3	70.1	84.1	72.7	51.9	89.3
85 and older	87.1	65.1	24.8	42.2	70.4	74.5	62.3	56.9	83.6
*p* value	0.00	0.31	0.00	0.00	0.00	0.00	0.00	0.44	0.00
Mother tongue language									
English	76.9	61.4	45.9	57.4	78.1	86.9	77.6	52.2	91.4
French	72.9	64.3	41.2	54.6	69.0	83.0	73.3	54.8	83.3
Other	68.6	67.9	33.8	51.6	77.8	86.8	75.8	57.2	87.5
*p* value	0.11	0.01	0.26	0.61	0.33	0.27	0.97	0.22	0.01
Immigration									
Born in Canada	76.8	62.2	46.2	55.7	76.1	86.3	76.3	52.5	90.2
Immigrant	71.3	63.6	37.0	58.6	79.8	86.5	78.6	55.2	89.3
*p* value	0.58	0.37	0.09	0.19	0.55	0.03	0.57	0.62	0.41
Income									
Less than $40,000	85.8	69.5	36.5	50.1	74.6	79.7	70.8	54.7	86.6
$40,000 to $69,999	70.9	61.3	41.5	55.5	73.1	85.0	76.9	51.9	86.8
$70,000 or more	72.8	61.8	54.6	65.5	80.6	92.5	87.3	52.2	94.0
Don’t know	84.1	68.9	26.0	44.5	73.9	74.9	57.7	56.8	85.0
Refused	73.6	59.2	44.5	55.2	78.4	87.9	75.4	53.1	91.5
*p* value	0.02	0.43	0.00	0.00	0.51	0.00	0.00	0.79	0.04
Education									
Did not graduate high school	87.7	56.7	26.0	42.3	60.7	71.9	60.9	52.1	76.9
Graduated high school	75.7	63.6	35.3	42.2	70.7	83.0	69.3	60.5	90.4
Some post-secondary	78.2	65.8	41.7	59.1	79.3	88.8	74	51.8	88.3
College or university graduation	72.8	62.4	50.3	62.2	81.3	89.4	82.3	51.4	92.5
*p* value	0.29	0.15	0.00	0.00	0.00	0.00	0.00	0.18	0.00

*Note: p* value is from univariate sub-group comparison (Pearson’s χ^2^).

### Annual Medication Review, Physical, and Vision Examination

**Figure 3:**
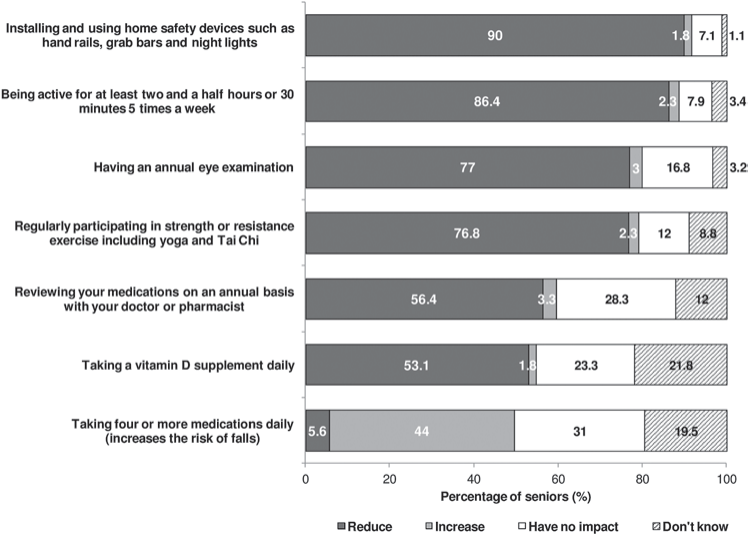
Perception of the impact of behaviours on the risk of falling

Fewer than half (44%) of respondents were aware that taking four or more medications daily can increase fall risk (Figure 3). This awareness decreased with age (65 to 74 years: 52%; >85 years: 25%) and lower income (>$70,000: 55%; <$40,000: 37%) but increased with higher levels of education (no high school graduation: 26%; college or university graduation: 50%; Table 3). Although 43 per cent of respondents reported taking four or more medications daily, 79 per cent of them reviewed the side effects with their health care provider in the past year (Table 4). Seventy-one per cent of respondents had a general physical examination less than one year ago (Table 4).

**Table 4:** Falls-prevention behaviours by socio-demographic characteristics (%)

Characteristic	General Physical Check-up <1 Year Ago	Eye Examination <1 Year Ago	Took ≥4 Medications Daily	Took ≥4 Medications Daily and Reviewed Side Effects with Health Provider	Met Aerobic Component of CPAG	Met Strength Component of CPAG	Participated in Stretching or Flexibility Exercise ≥1 per Week	Participated in Balance and Stability Exercise ≥1 per Week	Took a Vitamin D Supplement Daily	Daily Vitamin D Supplement and Recommended Calcium Intake (diet or supplementation)
Ottawa	70.7	75.4	43.1	78.7	51.0	37.6	40.0	36.3	45.9	18.5
Gender										
Male	70.2	74.2	46.2	80.2	59.5	43.1	39.4	26.8	34.8	10.5
Female	71.0	76.4	40.6	77.4	44.5	33.3	40.4	43.6	54.3	24.7
*p* value	0.79	0.31	0.19	0.57	0.00	0.00	0.77	0.00	0.00	0.00
Age (years)										
65 to 74	70.4	74.8	36.6	76.6	59.4	42.1	44.0	36.5	41.4	16.5
75 to 84	72.7	79.9	47.5	85.3	46.4	36.0	40.9	37.5	50.9	22.0
85 and older	67.2	67.6	57.6	71.1	29.5	23.4	22.5	32.8	51.3	18.4
*p* value	0.62	0.00	0.00	0.09	0.00	0.00	0.00	0.56	0.00	0.09
Mother tongue language										
English	70.5	77.5	45.2	79.8	51.4	37.5	40.5	36.0	46.5	19.3
French	73.8	66.8	41.2	76.6	46.7	35.9	34.3	38.5	42.7	17.0^a^
Other	69.2	71.2	31.3	70.6	53.2	39.7	42.9	36.3	44.9	15.1^a^
*p* value	0.00	0.01	0.06	0.06	0.58	0.85	0.39	0.88	0.64	0.48
Immigration										
Born in Canada	72.7	74.8	46.0	79.2	49.6	36.6	38.2	36.3	46.9	19.7
Immigrant	64.4	77.5	33.7	76.4	55.4	40.5	45.6	36.1	42.6	15.0
*p* value	0.04	0.14	0.00	0.80	0.13	0.31	0.05	0.95	0.56	0.10
Income										
Less than $40,000	68.5	67.6	43.4	72.6	39.1	25.1	35.1	42.7	41.1	21.6
$40,000 to $69,999	68.2	78.1	49.1	74.0	55.1	39.3	39.8	39.1	49.4	17.2
$70,000 or more	72.5	71.2	41.6	79.3	58.3	44.0	44.8	27.6	45.4	16.8
Don’t know	69.1	73.1	46.1	69.6	30.3	17.4	33.4	32.6	54.8	20.3^a^
Refused	72.2	81.3	39.6	73.5	52.7	41.6	40.1	38.9	44.5	18.9
*p* value	0.69	0.00	0.00	0.22	0.00	0.00	0.29	0.01	0.25	0.74
Education										
Did not graduate high school	70.9	64.9	47.5	75.3	32.0	26.3^a^	24.9	32.1	43.6	14.7
Graduated high school	72.1	76.7	45.3	73.3	43.6	21.2	31	30.2	48.5	18.3^a^
Some post-secondary	62.6	69.7	50.2	81.8	53.5	30.5	46.9	45.9	47.1	25.3
College or university graduation	71.8	77.8	40.7	80.0	56.4	45.7	43.9	37.2	45.3	17.9
*p* value	0.04	0.21	0.01	0.65	0.00		0.00	0.04	0.33	0.18

^**a**^ Coefficient of variation 16.6% to <33.3%.

*p* value is from univariate sub-group comparison (Pearson’s χ^2^).

CPAG = Canadian Physical Activity Guidelines for older adults

Seventy-seven per cent of respondents were aware that having an annual eye examination reduces fall risk. This awareness was higher among females and adults 65 to 74 years old, and increased with higher levels of education (no high school graduation: 61%; college or university graduation: 82%; Table 3). Seventy-five per cent of respondents had a vision examination less than one year ago. Respondents 85 years and older were less likely to have had their vision checked less than one year ago compared to those aged 75 to 84 years (Table 4).

### Physical Activity

Eighty-six per cent of respondents were aware that being active for 150 minutes (two and a half hours) a week reduces fall risk; 77 per cent were aware that regularly participating in strength or resistance exercises reduces fall risk (Figure 3). Awareness of these protective behaviours decreased with age (being active for two and a half hours/week reduces risk: at 65 to 74 years, 91% were aware and >85 years, 75% were aware; strength or balance exercise reduces risk: at 65 to 74 years, 83% were aware, and >85 years, 62% were aware) and increased with more education and income (Table 3). Fifty-one per cent of respondents met the aerobic component of CPAG-OA participating in at least 150 minutes of moderate- to vigorous-intensity physical activity. Thirty-eight per cent of respondents participated in strength activities at least twice a week, 40 per cent participated in stretching or flexibility exercises at least once per week, and 36 per cent participated in balance and stability exercises at least once per week. Males, those 65 to 84 years of age, those with highest education status, and those with higher household incomes were most likely to meet the guidelines for aerobic as well as strength activity (Table 4).

### Vitamin D and Calcium Intake

Half (55%) of respondents were aware that taking a vitamin D supplement daily can help prevent falls (Figure 3) – females were more likely than males to be aware of this (Table 3). Forty-six per cent of respondents took a supplement or multivitamin containing vitamin D daily – females and those aged 75 and older were more likely to report taking a vitamin D supplement daily (Table 4).

Nineteen per cent of respondents were taking a vitamin D supplement or multivitamin daily and consuming at least three servings of calcium-rich foods or two servings of calcium-rich food plus calcium supplementation daily as recommended in Canada’s Food Guide – females were more likely to meet this recommendation (Table 4).

### Home Safety Devices

Ninety per cent of respondents were aware that installing and using home safety devices reduces fall risk (Figure 3); this was lower among those aged 85 years and older and those who did not graduate high school (Table 3). Eighty-seven per cent of respondents with stairs at home had railings on one or both sides of the staircase; this was lower among those aged 65 to 74 years (Table 5). Half (52%) of respondents with mats or scatter rugs at home reported that all of them were secured to the floor (Table 5). Forty-four per cent of respondents regularly used extra night lighting to help them move about their homes at night (Table 5). Of respondents who used their home bath tub or shower, 71 per cent had a rubber bath mat or non-slip surface on the bath or shower floor – respondents aged 65 to 74 years and those with income of $70,000 or more were least likely to have one. Half (50%) of respondents who used their home bathtub or shower had grab bars or a rail installed; this was higher among females, those aged 75 years and older, those with income of less than $40,000, and those who did not graduate from high school. Nineteen per cent had a raised toilet or toilet seat – females and those aged 85 years and older were most likely to have one (Table 5).

**Table 5:** Falls prevention home safety strategies by socio-demographic characteristics (%)

Characteristic	Stairs Have Railings on One Or Both Sides	All Mats or Scatter Rugs Are Secured to the Floor	Regularly Use Extra Night-Lighting	Have a Rubber Bath Mat or Non-slip Surface on Bath/Shower Floor	Grab Bars or a Rail Installed in Bath/Shower	Raised Toilet Seat Installed
Ottawa	87.1	51.5	44.0	71.3	49.8	19.0
Gender						
Male	85.5	47.4	41.5	68.3	41.1	14.3
Female	88.4	54.9	45.8	73.7	56.5	22.7
*p* value	0.06	0.25	0.38	0.06	0.00	0.00
Age (year)						
65 to 74	83.5	52.3	43.3	66.7	37.7	16.6
75 to 84	91.3	53.5	45.0	75.5	61.5	17.6
85 and older	93.8	41.2	43.9	79.6	69.6	31.7
*p* value	0.00	0.35	0.00	0.00	0.00	0.00
Mother tongue language						
English	86.9	51.1	43.5	72.7	50.5	20.3
French	92.3	55.9	47.3	68.5	49	12.8
Other	84.3	50.4	44.0	64.9	46.1	17.0
*p* value	0.30	0.29	0.72	0.17	0.17	0.31
Immigration						
Born in Canada	87.2	52.9	45.1	72.6	50.9	19.6
Immigrant	86.9	47.1	40.4	67.1	46.5	17.3
*p* value	0.35	0.06	0.52	0.16	0.18	0.71
Income						
Less than $40,000	83.7	41.7	50.1	79.9	61.3	19.2
$40,000 to $69,999	91.9	56.4	41.7	73.7	49.7	17.0
$70,000 or more	82.9	51.0	42.1	61.6	39.2	16.8
Don’t know	88.4	52.5	45.8	75.5	71.6	27.3
Refused	88.8	52.6	43.4	72.4	48.2	20.4
*p* value	0.05	0.38	0.26	0.00	0.00	0.34
Education						
Did not graduate high school	86.5	49.1	49.8	80.3	67.1	25.1^a^
Graduated high school	90.3	42.7	45.1	72.6	57.8	20.1
Some post-secondary	92.0	47.0	51.9	68.5	54.8	21.2^a^
College or university graduation	85.5	54.5	41.4	69.7	43.9	17.4
*p* value	0.26	0.04	0.27	0.06	0.00	0.25

^**a**^ Coefficient of variation 16.6 to < 33.3%.

*p* value is from univariate sub-group comparison (Pearson’s χ^2^).

## Discussion

This survey is the first in Canada to examine the awareness and uptake of all of the six community-dwelling fall prevention recommendations and home environment modifications concurrently. To our knowledge, this is also the first Canadian study to report on the level of awareness among older adults that falls among people in their age group can be prevented – a message that public health organizations convey and use as the underpinning for fall prevention strategies (Public Health Agency of Canada [PHAC], 2014). Other studies have measured fear of falling but from an individual risk perspective (Scheffer, Schuurmans, van Dijk, van der Hooft, & de Rooij, 2008; Lee, Mackenzie, & James, 2008; Boyd & Stevens, 2009; Pearson, St-Arnaud, & Geran, 2014), including one national survey that found 34 per cent of Canadian adults aged 65 years and older were concerned about having a future fall (Pearson, St-Arnaud, & Geran, 2014). Our study found a higher perception of risk, with 76 per cent of respondents agreeing that falling is a concern for people their age. The difference suggests that older adults are generally aware of the risk of falls in their age group; however, they do not perceive the risk to the same extent individually.

Although perception of fall risk for older adults was high, results indicate that respondents were not aware of, and not taking, all steps to reduce the risk of falling. Only 44 per cent of respondents were aware that taking four or more medications increases risk of falling, and 56 per cent were aware that reviewing medications with their health care provider annually reduces risk. Encouragingly, a high proportion (79%) of the 43 per cent of respondents who took four or more medications daily had reviewed the side effects with a health care provider in the past year. One previous Canadian study of medication use among seniors living in private households found that 41 per cent of women and 29 per cent of men aged 65 years and older were taking four or more medications in the past month, but the study did not assess whether those taking multiple medications had reviewed them with a health care provider (Rotermann, 2006). Our findings indicate that adherence to this recommendation is high among one of the populations most at risk (multiple medication users). However, general awareness on the need to review medications with a health care provider to reduce fall risk is low, which suggests that universal health messaging may be warranted.

Our results found that 75 per cent of respondents had an eye examination less than one year ago, which was higher than the percentage found by a national study in 2003 (57%) (Rotermann, 2006). Although awareness that annual eye examination reduces fall risk was also high (77%), in Ontario routine eye examinations are covered by the Ontario Health Insurance Program once every 12 months for adults aged 65 years and older. Although we were unable to determine if the high proportion of those having an annual eye examination was influenced by high awareness or by universal coverage, both are plausible factors for the high level of adherence to this recommendation.

Physical activity plays an important role in preventing falls, but measurement is challenging because it is a multidimensional construct. Results from the 2012 and 2013 Canadian Health Measures Survey (CHMS) found that only 12 per cent of Canadian adults aged 60 to 79 years achieved the recommended aerobic component (at least 150 minutes of moderate- to vigorous-intensity activity per week) of the CPAG-OA when physical activity was directly measured (Statistics Canada, 2015). The CHMS is not directly comparable to our survey, but it suggests that our measurement of the proportion (51%) of older adults who met the aerobic activity component is likely an overestimate. It is widely known that self-reports are useful for gaining insight into a population’s physical activity levels, but they are known to overestimate true energy expenditure and physical activity because of recall and response biases (e.g., inaccurate memory, providing a socially desirable response). Although self-reported measurements do not capture the same amounts of physical activity as more direct measures (accelerometers, pedometers, etc.) (Prince et al., 2008), measuring direct physical activity was not within the capacity of this study. We recommend further research to examine differences between self-reported population levels of activity using the CHAMPS Physical Activity Questionnaire for Older Adults and direct measures.

With respect to the proportion of older adults meeting the strength component of the CPAG-OA (38%) and the proportion participating in strength and balance activities (36%) or stretching or flexibility exercises (40%) at least once per week, to our knowledge these components have not been measured for community-dwelling Canadian older adults. The validity and reliability of these measures in comparison to direct measurements is uncertain and could be an area for future research.

In Canada, only a few population health studies have examined the use of vitamin D supplements among older adults. The CHMS found that 34 per cent of Canadians took a supplement containing vitamin D in the past month and intake was higher among 40- to 79-year-olds (Janz & Pearson, 2013). Another Canadian study found that 60 per cent of British Columbian adults aged 50 years and older had used a vitamin D supplement in the past month (Green, Barr, & Chapman, 2010). In comparison, our results indicate that 46 per cent of respondents took a vitamin D supplement daily.

To our knowledge, there are no Canadian estimates of the use of home safety strategies among community-dwelling older adults. One federal report examined home modifications, but it was limited to older adults with disabilities (Human Resources and Skills Development Canada, 2011). In the United States, 78 per cent of adults aged 52 years and older had assistive features in the home, with common features including (1) railings in stairways (89%), (2) railings at the home entrances with steps (44%), (3) grab bars in the bath/shower (30%), (4) a seat for the bath/shower (27%), and (5) a raised toilet seat (15%) (Freedman & Agree, 2008). While the United States’ study population included both older and “near elderly” adults, our survey found similar proportions of the presence of stair railings (87%) and higher proportions of the installation of grab bars in the bath/shower (50%) and raised toilet seats (19%) – features whose installation in the home increases with respondent age.

Our findings indicate that, for the most part, there are inconsistencies between population level of awareness and adherence to fall prevention recommendations for community-dwelling older adults. High levels of awareness did not necessarily translate into high levels of behaviour, as demonstrated by the awareness and adherence to recommended physical activity guidelines and with recommended home safety modifications. On the contrary, low population levels of awareness did not always imply lower uptake as found with the recommendation regarding the taking of multiple medications and reviewing them with a health care provider. These results are not unexpected. Although it is generally thought that personal beliefs can influence behaviours, one behavioural change framework, the theory of planned behaviour, suggests that, in addition to beliefs and awareness, subjective norms and perceived behavioural control can shape intention along the pathway to behaviour change (Ajzen, 2002). External factors such as cost, access, and general health can also contribute to influencing behaviour, which may be the case with lower awareness but higher adherence to the recommendation related to medication review, as health care providers are expected to play a role in following the recommendation. However, we did not measure norms, perceived control, and other external factors or barriers for the lower levels in uptake. We suggest that future research might explore these reasons and examine whether perception of fall risk is associated with the apparent discrepancy.

The Public Health Agency of Canada (2014) has recommended a multifactorial and multisectoral approach to preventing falls, including public health interventions directed towards community-dwelling older adults and caregivers as well as collaboration between different sectors. Subpopulations that were found to have lower awareness of, and adherence to, the recommendations should be considered when planning equitable fall prevention interventions. Socio-demographic differences were found in some of the six fall prevention recommendations; however, further analysis is needed to control for relationships in these findings in order to inform tailored prevention approaches.

OPH’s Fall Prevention Approach focuses on five priority areas (OPH, 2015e): (1) monitoring and reporting falls-related statistics; (2) engaging key stakeholders to improve fall prevention health care services such as work with primary care on falls assessments and pharmacists and primary care to promote medication reviews; (3) expanding access to physical activity programming and products for older adults in the community with joint efforts with other sectors such as City of Ottawa recreation programs; (4) enhancing older adult environments to reduce falls by promoting environmental assessments and modifications to create safe environments and supporting a municipal Older Adult Plan that incorporates age-friendly initiatives (City of Ottawa, 2015); and (5) engaging older adults in fall prevention behaviours such as self-screening. OPH is an active member of the Champlain Regional Falls Prevention Program’s working group, which brings together hospitals, primary care, public health and community support services, and care access to identify and reduce the risk of falls (Champlain Local Health Integration Network, 2014). The findings from this study informed the direction for each of these priority areas and were further used to advocate for expansion in physical activity programming and collaboration.

### Challenges and Limitations

There were relevant challenges and limitations to the development of the survey. The primary challenge was to collect representative population-level data for older adults in Ottawa. A sampling frame of adults aged 65 years and older in Ottawa was not available; thus, substantial effort and cost was required to reach this target population through random-digit dialing, particularly among the oldest age group (85 years and older) who lived in private residences less frequently than 65- to 84-year-olds. As a result, the overall response rate seems low (23%); however, this includes a high number of non-responders who were likely not all age-eligible for inclusion. The generalizability of our results is limited to older adults in community dwellings who have and can use a landline telephone. With the trend towards the sole use of mobile phones, this limitation will become more apparent for studies of similar methodology. Another limitation is that the results may be biased towards those who chose to participate in the survey, and we were unable to collect any information about non-respondents.

Questionnaires targeted at older populations should be kept simple and concise so as not to cognitively overload the respondent. To mitigate this, we used some previously developed questions from well-established surveys and pilot-tested the full questionnaire on the target population. Still, not all questions in this survey have been assessed for their validity and reliability in this population.

Other challenges specific to collecting data on this population included respondent trust to provide confidential data such as income – in our survey, although the survey was introduced as being from a credible municipal organization with the offer of confidentiality, 32 per cent of respondents refused to provide their income. The survey was conducted in December, and the physical activity questions may be subject to seasonal variations (Shephard, 2003; Uitenbroek, 1993). The survey was limited to older respondents who could speak English or French. Because data were collected on adults living in their home, findings should not be extrapolated to older adults living in long-term care homes, nursing homes, and hospitals.

Although taking multiple medications concurrently has been established as a risk factor for falling, certain medications (e.g., psychotropic drugs) increase the risk of falling (de Jong, Van der Elst, & Hartholt, 2013). Specific medications were not captured in the survey, and the proportion of older adults at risk for falling because of medications is likely underestimated by focussing on multiple medication use only.

The questionnaire did not address previous history of falls or other co-morbidities associated with an increased risk of falls. These factors would likely affect the extent of awareness and behaviours related to reducing a risk of falls.

## Conclusion

Prevention approaches to reduce falls in community-dwelling adults aged 65 years and older are complex and multifaceted. However, essential to planning these approaches is an understanding of the levels of awareness and adherence to fall prevention recommendations. The Older Adults Fall Prevention Survey informed key priorities for planning fall prevention approaches. This survey tool can be used to assess fall prevention awareness and behaviours in other communities and the results may be applicable to similar Canadian settings.
